# Developing models for the diagnosing of ulcerative colitis and prognosis of anti-TNF-α non-response based on neutrophil extracellular trap-associated genes

**DOI:** 10.3389/fimmu.2025.1530508

**Published:** 2025-09-02

**Authors:** Jinyuan Ou, Linzhen Li, Fachao Zhi, Bing Huang, Xinmei Zhao

**Affiliations:** ^1^ Guangdong Provincial Key Laboratory of Gastroenterology, Institute of Gastroenterology of Guangdong Province, Department of Gastroenterology, Nanfang Hospital, Southern Medical University, Guangzhou, China; ^2^ Department of Radiology, The First Affiliated Hospital of Guangdong Pharmaceutical University, Guangzhou, China

**Keywords:** ulcerative colitis, neutrophil extracellular traps, anti-TNF-α, machine learning, predictive model

## Abstract

**Background:**

Neutrophil extracellular traps (NET) play a pivotal role in the pathogenesis of ulcerative colitis (UC) and may contribute to the impaired response to anti-tumor necrosis factor alpha (TNF-α) therapies. However, the functional implications of NET-associated genes in UC remain poorly understood. This study aims to identify key NET-associated molecular signatures in UC, develop diagnostic models based on NET-related biomarkers, and construct predictive models for response to anti-TNF-α therapies (infliximab and golimumab).

**Methods:**

NET-associated genes were obtained from the Kyoto Encyclopedia of Genes and Genomes, whereas UC-related gene expression datasets were retrieved from the Gene Expression Omnibus. Unsupervised consensus clustering based on NET-related genes was used to stratify patients with UC into molecular subtypes. The CIBERSORT algorithm and gene set variation analysis were employed to characterize immune cell infiltration and biological pathway activity across clusters. Hub genes were identified using weighted gene co-expression network analysis and machine learning algorithms. Spearman correlation analyses were performed to assess associations between hub genes, immune cell infiltration, and clinical disease activity. A diagnostic model for UC and a prognostic model for anti-TNF-α treatment response were developed using hub genes identified through least absolute shrinkage and selection operator regression.

**Results:**

Based on 33 NET-associated genes, patients with UC were stratified into two distinct molecular clusters (C1 and C2). Cluster C1 exhibited a pronounced NET signature, characterized by significantly elevated neutrophil infiltration (p < 0.001) and activation of inflammatory signaling pathways, including IL-2/STAT5, TNF-α/NF-κB, and IL-6/JAK/STAT3. Notably, C1 was associated with a significantly higher rate of non-response to anti-TNF-α therapy (57.4% vs. 22.0% in C2, p = 0.003). A diagnostic model for UC was constructed using five hub genes (FCGR3B, IL1RN, CXCL8, S100A8, and S100A9) derived from C1. Moreover, a predictive model for anti-TNF-α non-responsiveness, based on two hub genes (FCGR3B and IL1RN), was developed using a golimumab dataset and validated in two independent infliximab datasets.

**Conclusion:**

A distinct NET-associated cluster was identified among patients with UC, exhibiting non-responsiveness to anti-TNF-α treatment. Diagnostic and prognostic models based on NET-associated genes hold promise for guiding clinical treatment strategies.

## 1 Introduction

Ulcerative colitis (UC) is a chronic inflammatory disorder that affects the rectum and colon to varying degrees throughout a patient’s lifetime. As of 2023, an estimated five million cases exist globally, with incidence rates continuing to rise—particularly in developing regions, including Asia, where UC was once considered rare (1, 2). Although its etiology remains incompletely understood, genetic predisposition, immune dysregulation, and alterations in the gut microbiota are implicated in its pathogenesis (3). Current treatments range from 5-aminosalicylic acid (5-ASA) to biologic therapies, with anti-TNF agents (e.g., infliximab and golimumab) being a cornerstone of management. However, up to 40% of patients exhibit primary non-response to anti-TNF therapy, and many responders eventually develop secondary resistance (4). The mechanisms underlying treatment failure remain unclear, highlighting the need for molecular subtyping to stratify responders, identify novel targets, and guide personalized therapy.

Neutrophil extracellular traps (NET)—web-like structures released by activated neutrophils during inflammation— have been implicated in UC pathogenesis (5–7). Notably, successful anti-TNF therapy correlates with downregulation of NET-associated proteins and reduced NET formation (8). Recent studies from Chinese inflammatory bowel disease (IBD) centers further demonstrated an inverse relationship between NET levels, tissue infliximab concentrations, and mucosal healing (9, 10). However, the role of NET-associated genes in UC heterogeneity and anti-TNF resistance is poorly characterized.

This study demonstrates a notable correlation between NETs and the heterogeneity of treatment response among patients with UC. By analyzing NET-associated gene expression profiles, patients with UC were classified into two distinct clusters: C1 and C2. The C1 cluster exhibited enhanced NET-related features, severe immune phenotype, heightened activation of immune pathways, along with a greater likelihood of non-response to anti-TNF-α therapy. Subsequently, machine learning was employed to identify five hub genes (FCGR3B, IL1RN, CXCL8, S100A8, and S100A9) implicated in disease onset, progression, and neutrophil extracellular trap formation. Finally, we developed models for diagnosing UC and predicting non-response to anti-TNF therapy. These models may assist in the diagnosis and treatment of UC.

## 2 Methods

### 2.1 NET-associated genes from the Kyoto Encyclopedia of Genes and Genomes database

NET-associated gene data were retrieved from the Kyoto Encyclopedia of Genes and Genomes Database, a comprehensive database that integrates genomic, chemical, and systemic functional information. It helps researchers understand biological processes and interactions by providing detailed pathways and networks involving various molecules (11). A total of 192 NET-associated genes (hsa04613) were obtained for analysis.

### 2.2 Data collection from the Gene Expression Omnibus database

**Figure 1:**
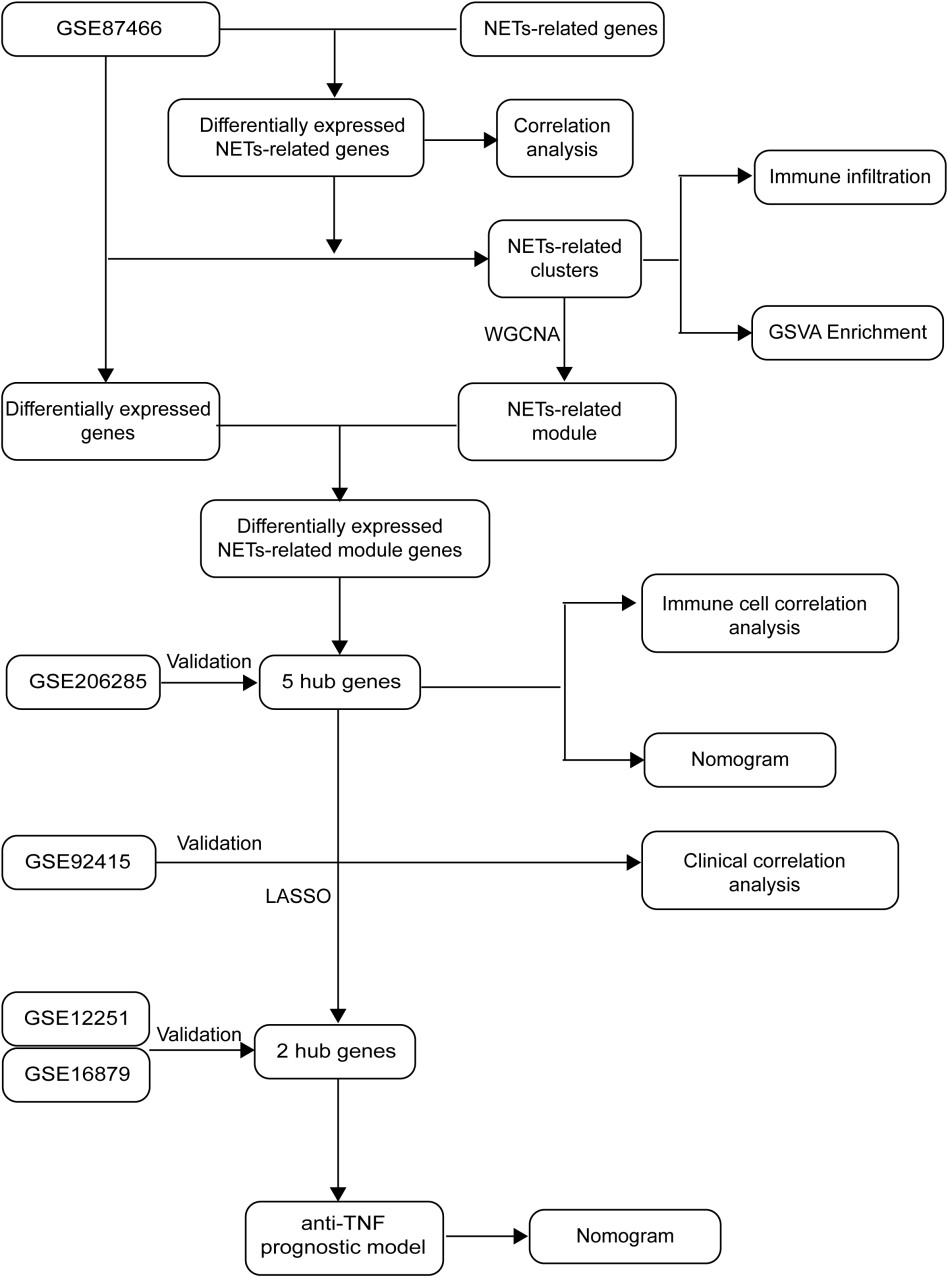
Study flowchart.

**Figure 1** depicts the flowchart of our project, which draws on the methodology of Zheng et al. (12). Relevant datasets of intestinal mucosal biopsies from patients diagnosed with UC were sourced from the Gene Expression Omnibus database. The datasets encompassed GSE87466 (87 UC samples, 21 healthy controls), GSE206285 (551 UC samples, 18 controls), GSE92415 (61 UC samples with positive response to golimumab treatment, 48 with no response), GSE12251 (12 UC samples with a favorable response to infliximab, 11 with no response), and GSE16879 (16 UC samples with a favorable response to infliximab, 32 with no response).

### 2.3 Analysis of differential expression and co-expression patterns of NET-associated genes

The Mann-Whitney U test was utilized to examine the variations in NET-associated genes between patients with UC (87 samples) and healthy controls (21 samples) within the GSE87466, using a significance level of p < 0.0001 as the threshold. Subsequently, the “cor” function was utilized to calculate the Pearson correlation coefficients for the identified NET-associated genes.

### 2.4 Consensus clustering

Using the R package “ConsensusClusterPlus” (13), we performed consensus clustering to distinguish NET-associated subtypes based on the 33 differentially expressed genes. The optimal cluster number was determined beforehand. To test the quality of clustering, we used the R package “FactoMineR” to visualize differences among the clusters via principal component analysis.

### 2.5 Gene set variant analysis

To characterize the molecular biological differences between the two UC clusters, we applied the “clusterProfiler” (14) package to compute signaling pathway variation scores for each gene set sourced from the Molecular Signatures Database (MSigDB). The “limma” (14) was used to identify the differential signatures among various groups.

### 2.6 Immune infiltration analysis

CIBERSORT (15) was employed to measure the levels of 22 immune cell types present in the colonic mucosa of individuals with UC and healthy individuals for comparison. To validate the distinctions between the two groups, we employed Student’s t-test, with the findings illustrated using the “ggboxplot” function in R.

### 2.7 Weighted gene co-expression network analysis for identifying NET-associated UC hub genes

To investigate NET-associated genes in UC, we applied the “WGCNA” R package (16) to conduct a WGCNA. As the initial clustering of UC samples was based on NET-associated genes, the module exhibiting the strongest association with UC was designated as the NET-associated module. To identify differentially expressed genes in patients with UC and controls, we applied the “limma” package to the GSE87466 dataset. A log fold change (logFC) threshold with an absolute value of 2 and a p-value threshold of 0.05 were set for this analysis. By finding the intersection of genes differentially expressed and those within the NET-associated module, we identified the differentially expressed NET-associated module genes. These genes hold important value for future research as they are implicated in the pathogenesis of UC and associated with NET.

Machine learning algorithms (17) automatically evaluates the regulatory weights between genes through model training and thus are widely used in identifying hub genes. To determine which algorithm exhibits superior classification performance, we used three machine learning algorithms: the generalized linear model (GLM), support vector machine (SVM), and random forest (RF). The receiver operating characteristic curve was used to evaluate the classification performance of machine learning. Subsequently, the feature plot was utilized to identify hub genes associated with the onset and progression of UC, as well as those associated with NET.

### 2.8 Building and evaluating the diagnosis of UC

The GSE87466 dataset was split into a training set, comprising 70% of the data, and a validation set, consisting of 30%, based on these five genes. A diagnostic model for ulcerative colitis was then constructed using the training data and evaluated using the independent GSE206285 dataset. Model performance was visualized using a nomogram.

### 2.9 Correlation analysis between hub genes and immune cell abundance and disease activity index

To further explore the impacts of hub genes, we analyzed their associations with immune cell infiltration and Mayo scores using the Spearman correlation analysis method. The Mayo score, a clinically relevant system for evaluating UC severity, integrates endoscopic observations and clinical symptoms (18).

### 2.10 Developing a predictive model for identifying non-responders to anti-TNF-α therapy

To examine the effectiveness of golimumab treatment across different UC subgroups, we stratified patients in the GSE92415 dataset into two distinct clusters: C1 and C2, according to the five hub genes associated with NET. The chi-square test was used to examine whether there is a difference in the ineffectiveness of golimumab treatment between the two clusters. To further elucidate the genetic markers associated with diverse responses to anti-TNF-α therapies, we conducted least absolute shrinkage and selection operator regression analysis, using the previously identified hub genes. Thus, we developed a predictive model capable of identifying patients who may not respond to golimumab. The R package “glmnet” was employed. Additionally, we created a nomogram using the R package “rms” to facilitate clinical application of our model. The model’s predictive prowess was gauged using the area under the curve (AUC), with its reliability validated using the GSE12251 dataset.

## 3 Results

### 3.1 Differential expression analysis identifies 33 NET-associated genes in UC

**Figure 2:**
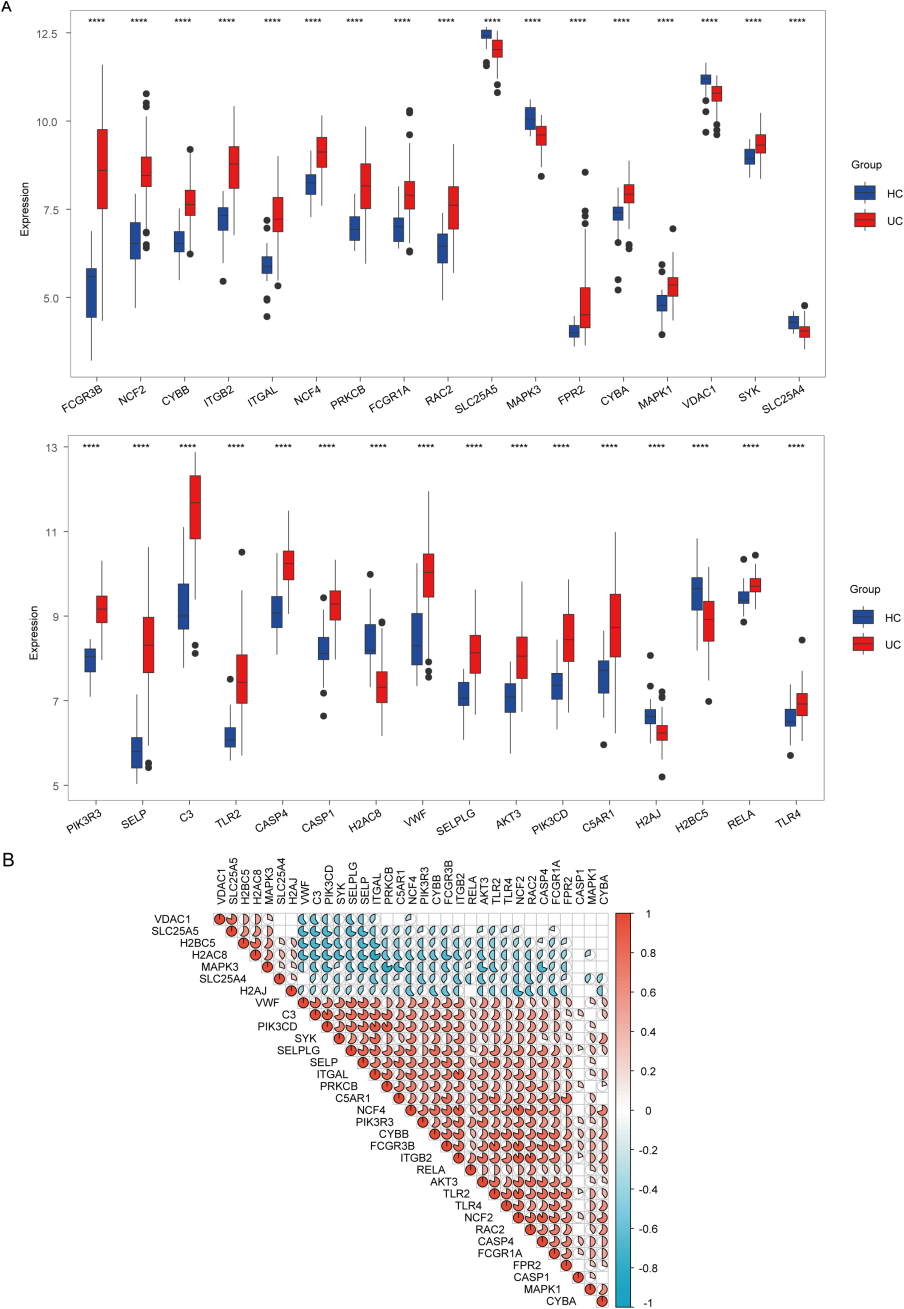
Analysis of expression and correlation of neutrophil extracellular traps (NET)-associated genes in ulcerative colitis (UC) dataset GSE87466. **(A)** Expression difference of NET-associated genes in patients with UC and healthy controls (HC). **(B)** Correlation of differentially expressed NET-associated genes. Red hues denote positive correlations (r > 0), whereas blue hues indicate negative correlations (r < 0), and the color depth represents correlation strength. The gradient from red to blue illustrates the transition from positive to negative correlation values. **** indicates p < 0.001.

To explore the involvement of NET in the onset and progression of UC, we retrieved 192 genes from the database of Kyoto Encyclopedia of Genes and Genomes. These genes were then compared between the mucosal tissues of the colon in patients with UC and those of healthy controls. Of these, 33 genes exhibited statistically significant differential expression (p < 0.0001): *Fc fragment of IgG receptor IIIb* (*FCGR3B*), sp*leen associated tyrosine kinase* (*SYK*), *mitogen-activated protein kinase 1* (*MAPK1*)*, mitogen-activated protein kinase 3* (*MAPK3*)*, cytochrome b-245 beta chain* (*CYBB*), *cytochrome b-245 alpha chain* (*CYBA*), *neutrophil cytosolic factor 2* (*NCF2*), *neutrophil cytosolic factor 4* (*NCF4*), *rac family small GTPase 2* (*RAC2*), *voltage dependent anion channel 1* (*VDAC1*), *volute carrier family 25 member 4* (*SLC25A4*), *solute carrier family 25 member 5* (*SLC25A5*), *Fc fragment of IgG receptor Ia* (*FCGR1A*)*, integrin subunit beta 2* (*ITGB*2), *integrin subunit alpha L* (*ITGAL*), *protein kinase C beta* (*PRKCB*), *formyl peptide receptor 2* (*FPR2*), *phosphoinositide-3-kinase catalytic delta* (*PIK3CD*), *phosphoinositide-3-kinase regulatory subunit 3* (*PIK3R3*), *AKT serine/threonine kinase 3* (*AKT3*), *RELA proto-oncogene* (*NF-κB subunit*) (*RELA*)*, complement C3* (*C3*), *complement C5a receptor 1* (*C5AR1*)*, toll-like receptor 2* (*TLR2*)*, toll-like receptor 4* (*TLR4*), *Von Willebrand factor* (*VWF*), *selectin P* (*SELP*), *selectin P ligand* (*SELPLG*), *caspase 4* (*CASP4*), *caspase 1* (*CASP1*)*, H2A clustered histone 8* (*H2AC8*), *H2A histone family member J* (*H2AJ*), and *H2B clustered histone 5* (*H2BC5*) (**Figure 2A**). These differentially expressed NET-associated genes were subsequently utilized for co-expression analysis. The findings uncovered regulatory interactions among the NET-associated genes, with the majority displaying positive regulation (**Figure 2B**).

### 3.2 Cluster C1 identification as a NET-associated subtype

**Figure 3:**
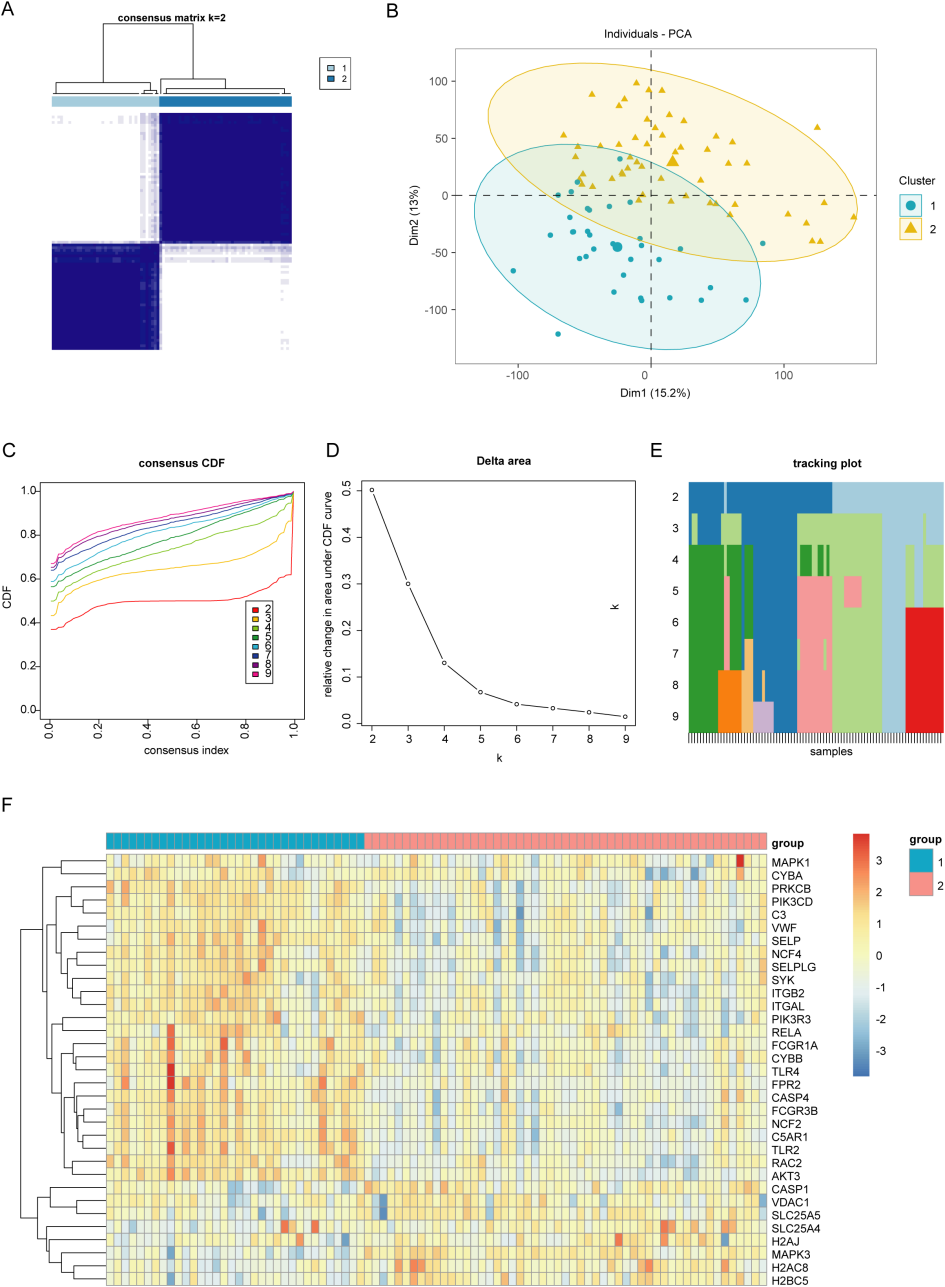
Identifying different clusters of ulcerative colitis (UC). **(A)** Consensus matrix of UC samples in GSE87466 (k = 2). **(B)** Principal Component Analysis plot exhibiting the degree of distinction between different UC clusters (C1 and C2). **(C)** Consensus cumulative distribution function (CDF) plot showing the area under the curve for k = 2–9. **(D)** Relative change in the area under the CDF curve. **(E)** Tracking plot exhibiting the sample subtypes for different values of (k). The color block represents the subtype number of the sample. **(F)** Heatmap showing the expression of neutrophil extracellular traps (NET)-associated genes between two UC clusters (C1 and C2).

To better understand the role of NET in UC, we categorized UC samples into two different clusters: C1 and C2, based on differentially expressed NET-associated genes (**Figure 3A**). The principal component analysis (PCA) plot demonstrates the clear separation between the two clusters, suggesting that these groups exhibit distinct characteristics (**Figure 3B**). The optimal number of subtypes was set at 2, based on the objective of the research, consensus matrix diagrams, cumulative distribution function (CDF) charts, percentage changes in areas beneath the CDF curve, and trajectory plots (**Figures 3C–E**). Then, we compared the expression levels of the differentially expressed NET-associated genes between the two clusters. *CASP1*, *VDAC1*, *SLC25A5*, *SLC25A4*, *H2AJ*, *MAPK3*, *H2AC8*, and *H2BC5* were downregulated in cluster C1, whereas the expression of other NET-associated genes, linked to the positive modulation of NET, was upregulated (**Figure 3F**). Thus, C1 was identified as a NET-associated cluster.

### 3.3 Immune cell infiltration signatures and gene set variation analysis of NET-associated cluster C1

**Figure 4:**
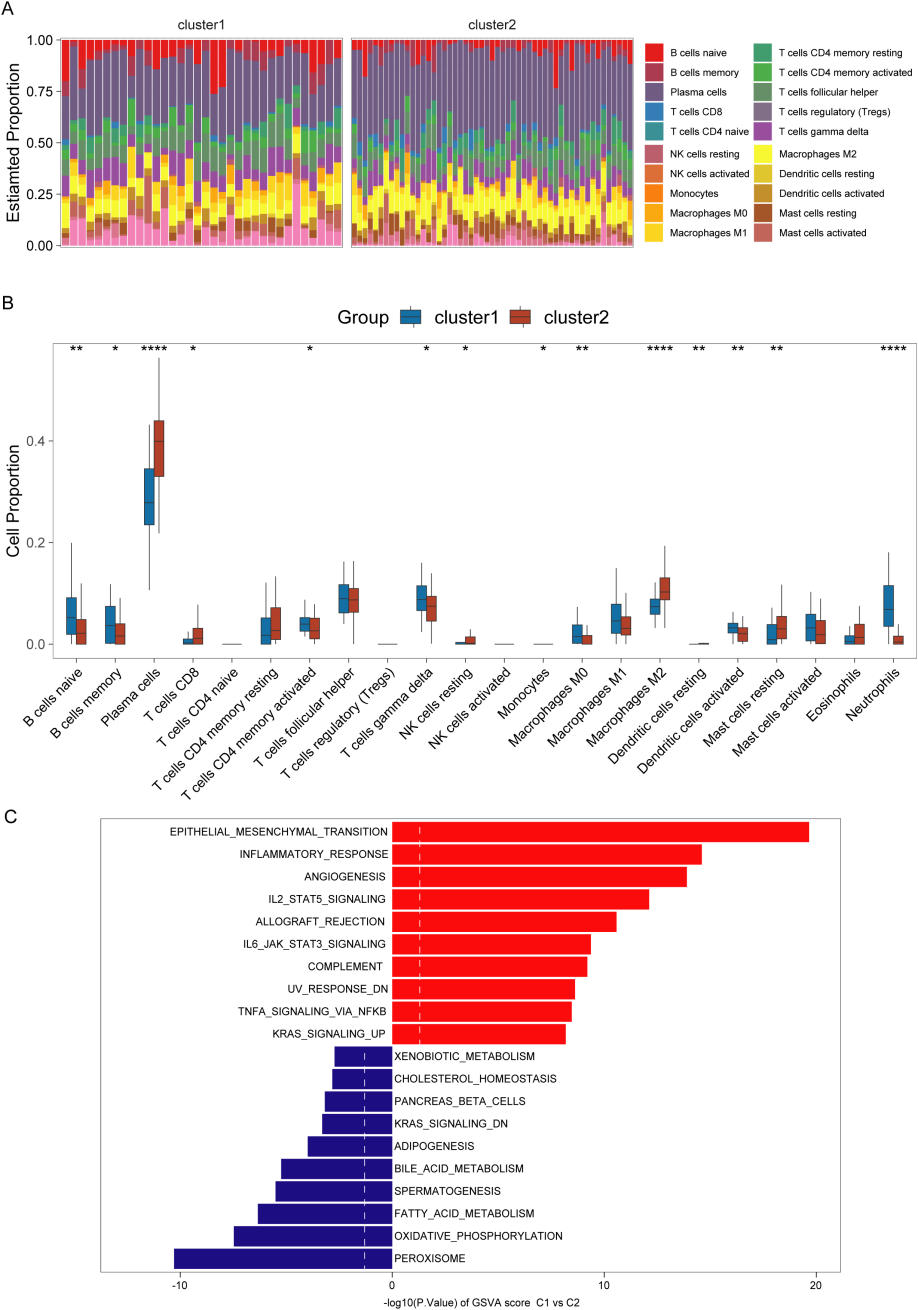
Immunological and pathway differences among different clusters of ulcerative colitis (UC). **(A, B)** Immune infiltration abundance in UC clusters (C1 and C2). **(C)** Gene set variation enrichment analysis of C1 and C2 clusters in UC (C2 cluster as control). *,**,**** indicate p < 0.05, p < 0.01, and p < 0.001.

Immune cell infiltration analysis reveals that cluster C1 was enriched in neutrophils, activated dendritic cells, M0 macrophages, memory B cells, activated memory CD4^+^ T cells, and gamma delta cells. Conversely, cluster C2 exhibited higher levels of M2 macrophages, resting NK cells, resting dendritic cells, plasma cells, CD8^+^ T cells, and resting mast cells (**Figures 4A, B**). To uncover the molecular basis for the differential immune infiltration patterns observed between the two clusters, we conducted gene set variant enrichment analysis between the two clusters. We found that epithelial-mesenchymal transition, inflammatory response, and angiogenesis exhibited enhanced activity within the C1 cluster. Meanwhile, the C2 cluster was correlated with metabolic pathways encompassing peroxisome activity, oxidative phosphorylation, and fatty acid catabolism (**Figure 4C**).

### 3.4 WGCNA analysis for identifying differentially expressed NET-associated module genes

**Figure 5:**
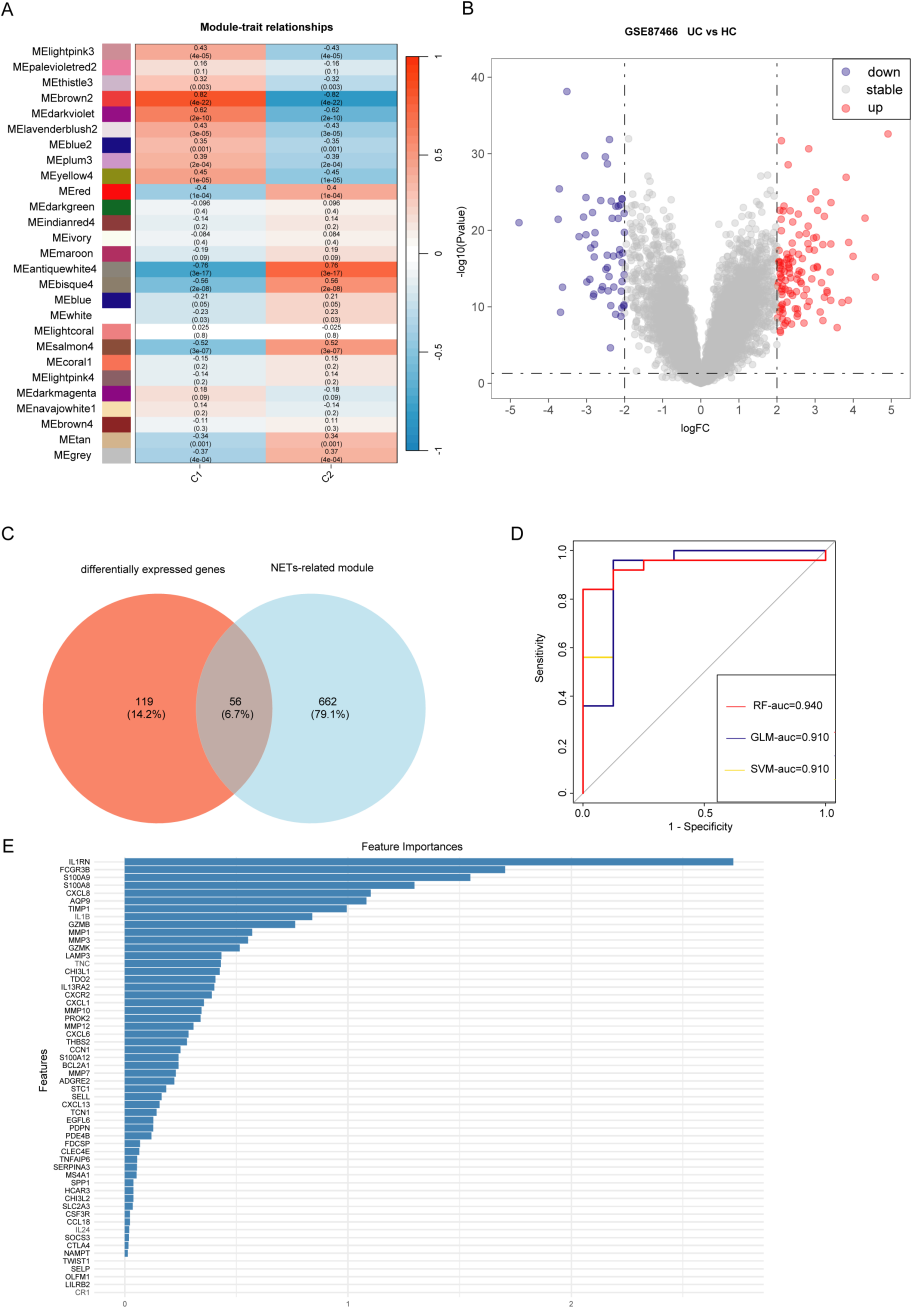
Identification of genes involved in the development of ulcerative colitis (UC) and neutrophil extracellular traps (NET). **(A)** Weighted gene co-expression network analysis (WGCNA) module trait for C1 and C2 clusters. **(B)** Volcano plot displaying gene expression differences between UC samples and healthy control samples. **(C)** Intersection of differentially expressed genes between UC samples (differentially expressed genes) and control samples with characteristic module genes of UC clusters (Module). **(D)** Receiver operating characteristic (ROC) curves showing the high diagnostic performance of three deep learning models for UC: Random forest (RF), Generalized linear model (GLM), and Support vector machine (SVM). **(E)** Top five significant genes (IL1RN, FCGR3B, S100A8, S100A9, CXCL8) as hub genes identified using the Random Forest (RF) algorithm.

WGCNA was conducted on data from patients with UC, revealing that the brown module “ME brown2” exhibited the strongest association with the C1 cluster (**Figure 5A**, **Supplementary Figures 1A–C**). This indicated that the ME brown2 module was the characteristic module of the NET-associated cluster C1. In other words, the genes included in this module were the most distinguishing factors between the C1 and C2 clusters. As C1 and C2 clusters were differentiated based on differences in NET-associated genes, this suggested a correlation between the genes in the ME brown2 module and NET. Therefore, we designated this brown2-module as the NET-associated module. **Figure 5B** shows that by setting the threshold at an absolute logFC value of 2 and a p-value of 0.05, we obtained 175 differentially expressed genes. By overlapping the genes in the NET-associated module with differentially expressed genes, we identified 56 module genes specifically associated with NETs that are also differentially expressed (**Figure 5C**). These genes were associated with NETs and implicated in the progression of the disease, leading to their definition as differentially expressed NET-associated module genes.

### 3.5 Identifying hub genes and developing a model for diagnosing UC

**Figure 6:**
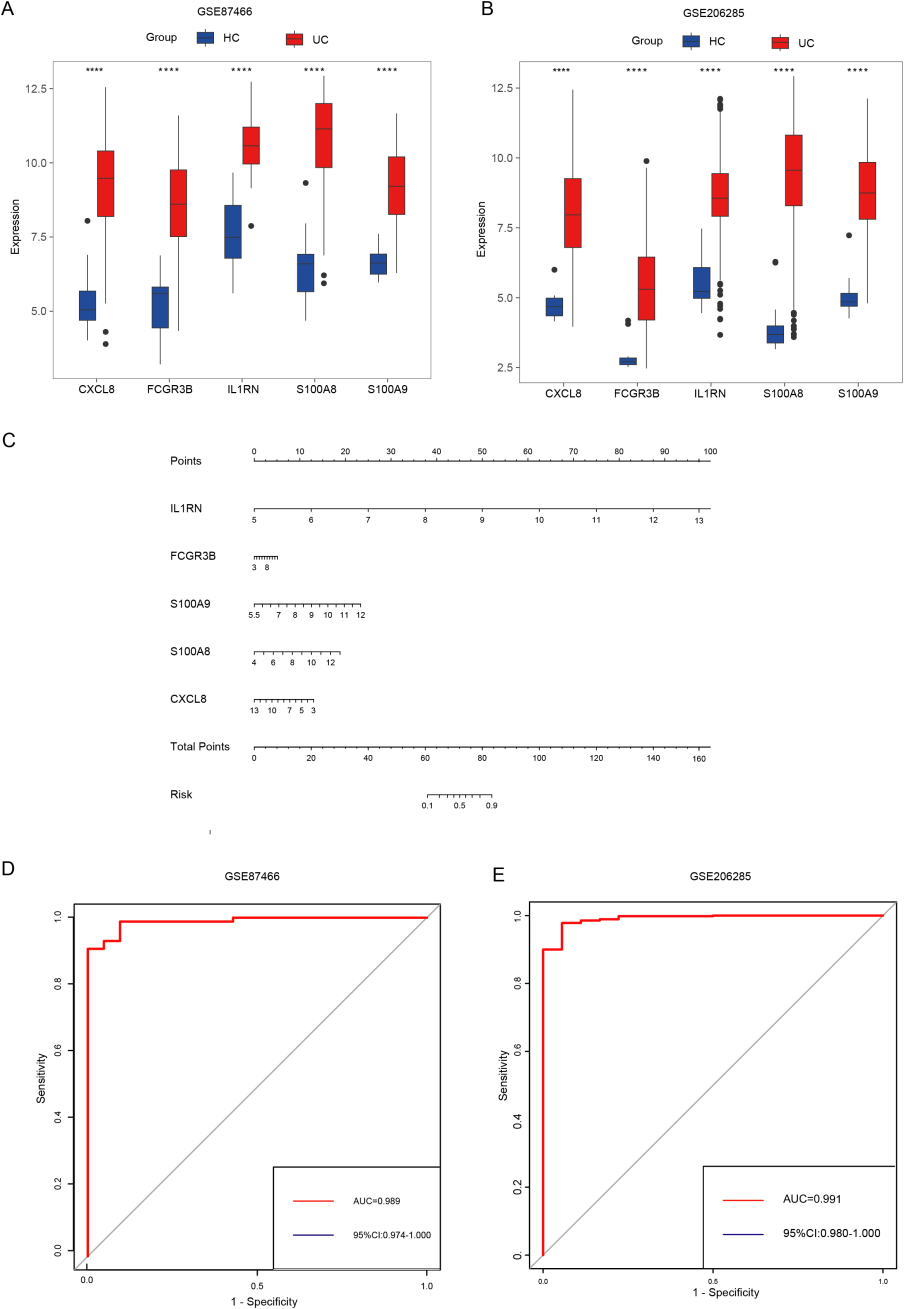
Identifying hub genes and constructing diagnostic models. **(A, B)** Differential expression of hub genes between patients with UC and healthy controls in GSE87466 **(A)** and GSE206285 **(B)**. **(C)** Nomogram for the diagnosis of UC. **(D, E)** The diagnostic ability of hub genes in GSE87466 **(D)** and GSE206285 **(E)**. **** indicates p < 0.001.

Machine learning algorithms are widely used in identifying hub genes. According to the differentially expressed NET-associated module genes, the RF algorithm demonstrated a higher efficiency in distinguishing patients with UC (**Figure 5D**). Moreover, using the RF algorithm, we identified interleukin 1 receptor antagonist (IL1RN), Fc fragment of IgG receptor IIIb (FCGR3B), S100 calcium binding protein A9 (S100A9), S100 calcium binding protein A8 (S100A8), C-X-C motif chemokine ligand 8 (CXCL8) as the five most prominent hub genes (**Figure 5E**). In the GSE87466 and GSE206285 datasets (**Figures 6A, B**), these five hub genes were upregulated in patients with UC. Using these identified hub genes as a foundation, a diagnostic nomogram for UC was developed (**Figure 6C**). The model’s exceptional performance was demonstrated in GSE87466 and was further externally confirmed in GSE206285 (**Figures 6D, E**).

### 3.6 The hub genes are associated with pro-inflammatory immune cell infiltration and severe disease activity

**Figure 7:**
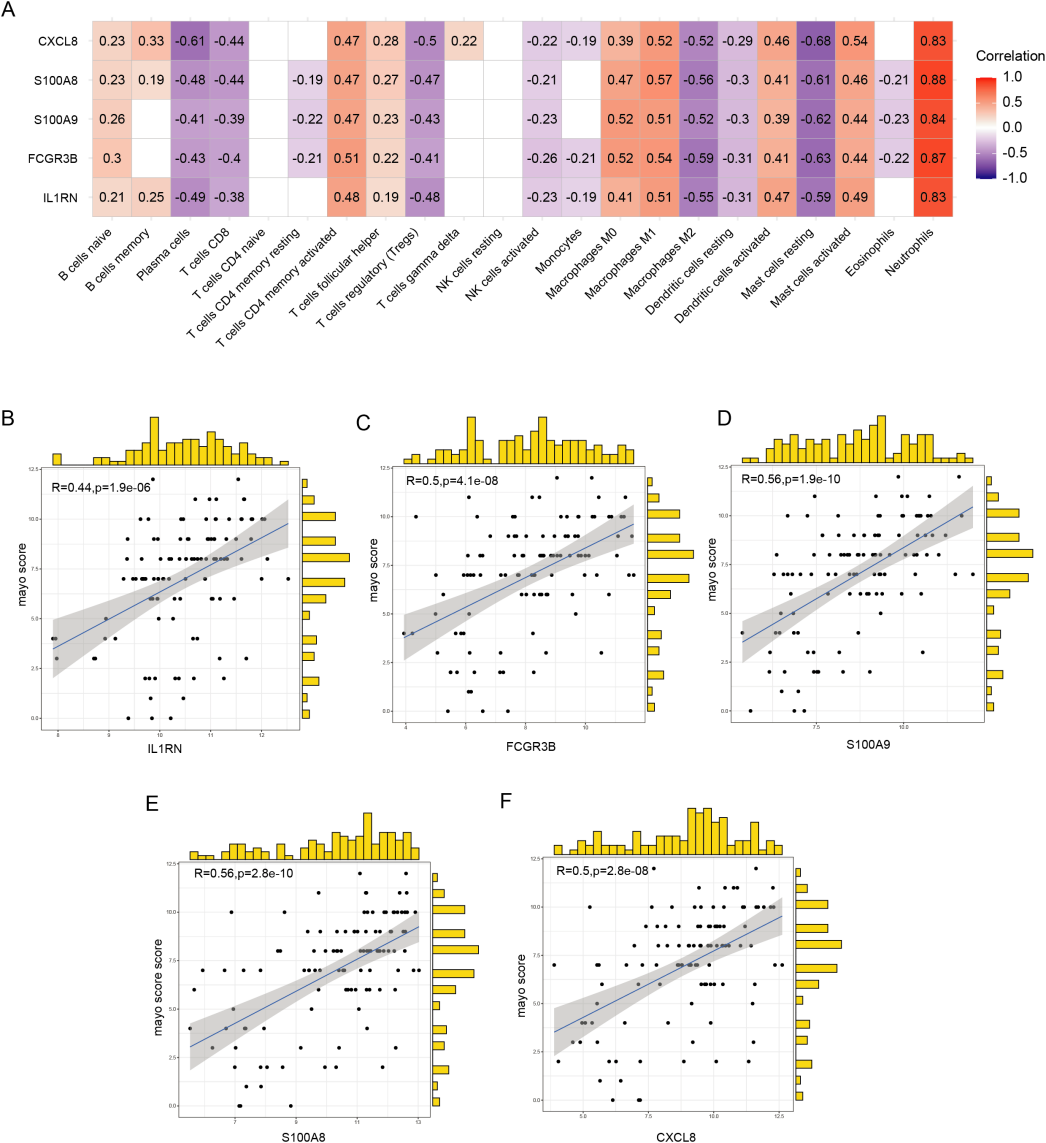
Correlation between hub genes, immune infiltration, and clinical severity. **(A)** Correlation between hub genes and the abundance of immune cell infiltration. **(B–F)** Correlation of five hub genes with Mayo scores.

The hub genes, namely IL1RN, FCGR3B, S100A9, S100A8, and CXCL8, exhibited a positive correlation with the abundance of neutrophils, activated mast and dendritic cells, M1 macrophages, M0 macrophages, activated CD4^+^ memory T cells, follicular helper T cells, and naïve B cells. Conversely, these hub genes exhibited a negative correlation with the abundance of resting mast cells, M2 macrophages, resting dendritic cells, activated NK cells, regulatory T cells (Tregs), CD^+^ T cells, and plasma cells (**Figure 7A**). Among the detected hub genes, S100A8 demonstrated the most pronounced positive correlation with neutrophils, whereas FCGR3B displayed the most significant negative association with M2 macrophages (**Figure 7A**). Analysis of the correlation between expression levels of hub genes and clinical severity showed that these genes demonstrated a positive association with Mayo scores (**Figures 7B–F**).

### 3.7 Construction and validation of predictive model for anti-TNF-α non-response based on hub genes

**Figure 8:**
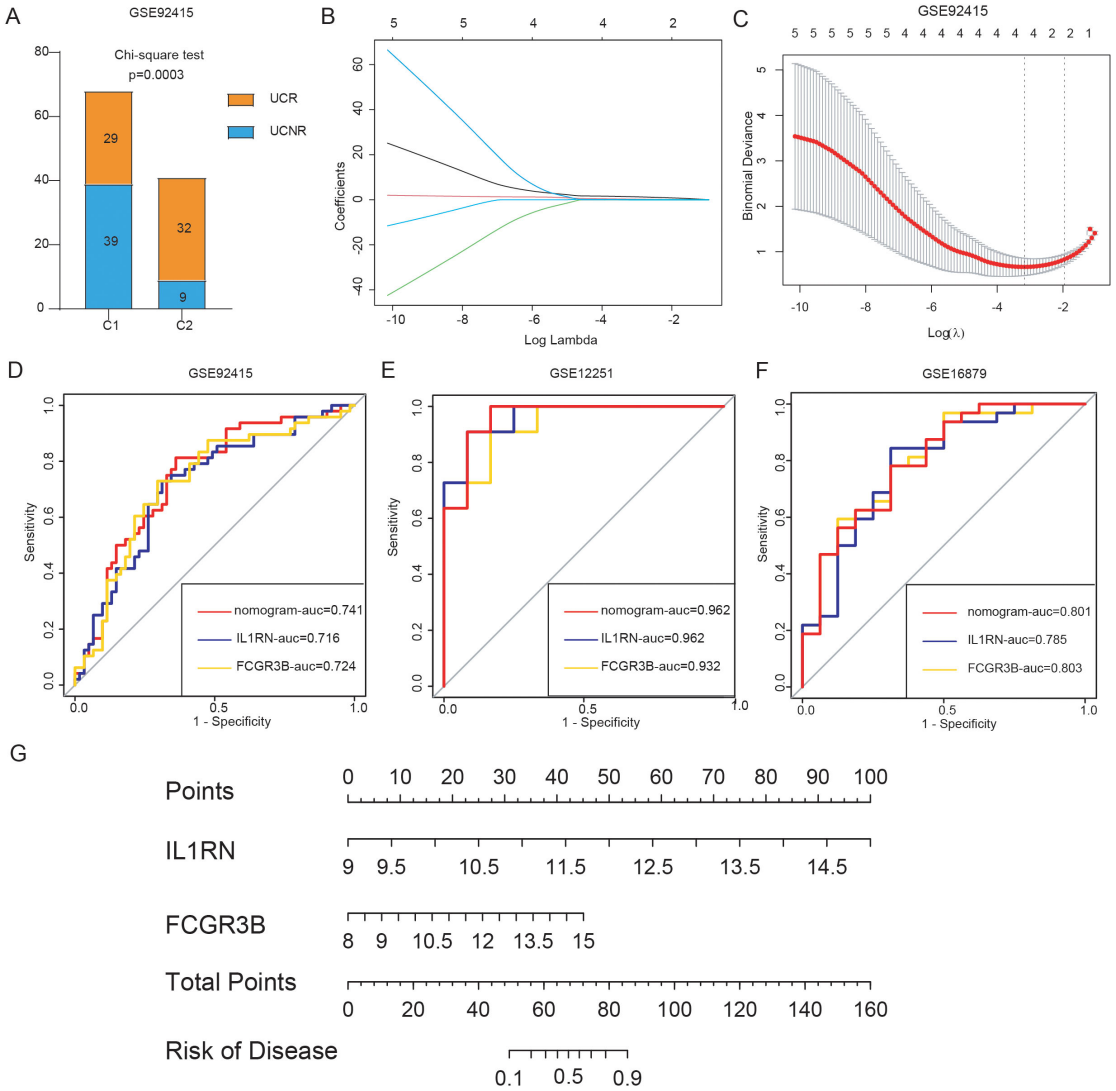
Construction and validation of the predictive model for anti-tumor necrosis factor alpha (anti-TNF-α) non-response. **(A)** Treatment response to golimumab among patients with ulcerative colitis (UC) in the GSE92415 dataset. **(B, C)** Cross-validation for optimal parameter tuning using least absolute shrinkage and selection operator regression analysis. **(D)** Receiver operating characteristic (ROC) curves evaluating the predictive performance of IL1RN, FCGR3B, and the nomogram for identifying golimumab non-responders in GSE92415. **(E, F)** ROC curves assessing the ability of IL1RN, FCGR3B, and the nomogram to predict infliximab non-response in external validation datasets GSE12251 and GSE16879. **(G)** Nomogram for predicting anti-TNF-α non-response in patients.

To investigate potential differences in golimumab response among distinct UC clusters, we classified patients in the GSE92415 dataset into two separate clusters: C1 and C2, using five hub genes. Our analysis revealed that 29 individuals with UC showed a positive response to golimumab treatment, whereas 39 individuals with UC did not respond in cluster C1. Conversely, within the C2 cluster, 32 patients responded positively to the treatment, whereas 9 patients did not respond (Chi-square test, p = 0.0003) (**Figure 8A**). This suggested a greater probability of patients in the C1 cluster not responding to golimumab treatment. To identify the genetic factors associated with differential responses to golimumab, we conducted least absolute shrinkage and selection operator regression analysis (**Figures 8B, C**). This analysis revealed that IL1RN and FCGR3B were associated with non-response to golimumab. Both genes exhibited strong predictive capabilities: IL1RN attained an AUC of 0.716, and FCGR3B achieved an AUC of 0.724 (**Figure 8D**). Utilizing IL1RN and FCGR3B, we constructed a model for identifying non-responders to golimumab, which achieved an AUC of 0.741. To confirm the robustness of our findings, we validated the results in two independent cohorts: GSE12251 and GSE16879. The diagnostic performance of our model for predicting non-response to infliximab achieved AUCs of 0.962 (GSE12251) and 0.801 (GSE16879) (**Figures 8E, F**). Finally, we presented this model as a nomogram for ease of interpretation (**Figure 8G**).

## 4 Discussion

UC is a complex, chronic inflammatory condition of the intestinal tract, in which establishing a definitive diagnosis remains challenging due to its heterogeneous clinical manifestations and the need to differentiate it from other gastrointestinal diseases (19). Furthermore, up to 40% of patients with UC do not respond to anti-TNF therapy (4). This high rate of therapeutic failure reflects the underlying gap in our comprehensive understanding of UC pathogenesis. Thus, it is imperative to investigate treatment responses—particularly to anti-TNF-α therapy—from diverse pathophysiological perspectives and to identify patients at risk of non-response. Molecular subtyping of UC offers a promising strategy to stratify non-responders and facilitate the development of novel therapeutic approaches.

In this study, we identified distinct expression profiles of multiple NET-associated genes in UC, using a stringent significance threshold of p = 0.0001 to prioritize genes of high relevance. Several of these genes, including CASP1 (20) and TLR4 (21), have been previously implicated in the initiation and progression of UC. Based on NET-associated gene expression, we classified patients into two molecular clusters.

Immune cell infiltration analysis revealed that the C1 cluster exhibited higher abundances of neutrophils, activated dendritic cells, M0 macrophages, memory B cells, activated memory CD4^+^ T cells, and gamma delta T cells. In comparison to C2, the C1 cluster also showed increased activation of several key immune pathways, including IL-2/STAT5, TNF-α/NF-κB, and IL-6/JAK/STAT3. Each of these pathways has been mechanistically linked to NET formation: IL-2 induces reactive oxygen species production and autophagy, thereby promoting NET formation (22); TNF-α directly stimulates NET release from neutrophils (8); and IL-6–mediated JAK/STAT3 signaling prolongs neutrophil survival and enhances functional capacity, indirectly facilitating NET formation (23). Taken together, these findings indicate that the C1 cluster represents a hyperinflammatory UC subtype with elevated NET activity, which may underlie its more severe immune phenotype and higher likelihood of resistance to anti-TNF-α therapy.

Further analysis revealed that the C1 cluster, which is closely linked to NET, exhibited a significantly higher non-response rate to golimumab treatment, suggesting a potential role of NET in mediating therapeutic resistance. There are two reasons why golimumab was chosen for further analysis. Firstly, it has been observed that NET are associated with other anti-TNF agents, such as infliximab and adalimumab (24). The selection of golimumab for further analysis can enhance the comprehensive understanding of the relationship between NET and anti-TNF therapy. Secondly, due to practical reasons, GSE92415 is the only dataset we could find that includes both disease activity scores and anti-TNF-α treatment healing data before anti-TNF therapy. Evaluating the correlation between the selected NET-related hub genes and disease activity, as well as assessing the responsiveness of hub genes from different clusters to anti-TNF-α therapy, can strengthen the credibility of the conclusions and reinforce the connection between NET and the efficacy of anti-TNF treatment. Similar to our results, a study by Monteleone et al. reported that successful infliximab treatment in patients with UC correlated with the downregulation of NET-associated proteins and suppression of NET formation (8). Similarly, two studies from Chinese inflammatory bowel disease centers demonstrated that NET levels were inversely correlated with tissue infliximab concentrations and mucosal healing outcomes in Crohn’s disease, an IBD subtype with pathophysiological similarities to UC. These findings collectively support a potential mechanistic link between NET activity and reduced therapeutic efficacy of anti-TNF agents in UC.

One proposed mechanism involves elevated neutrophil elastase activity within the UC mucosa, which may proteolytically degrade anti-TNF biologics, contributing to treatment failure (24). Moreover, NET are known to upregulate proteolytic enzymes such as matrix metalloproteinases, which further facilitate the degradation of anti-TNF-α agents and compromise therapeutic effectiveness (24).

During our investigation of NET-associated gene expression, we observed upregulation of IL1RN, FCGR3B, S100A9, S100A8, and CXCL8 in patients with UC. Among these, IL1RN and FCGR3B emerged as strong predictors of non-response to golimumab. This association was further validated in an infliximab non-response dataset, reinforcing the link between NET activity and resistance to anti-TNF therapy.

FCGR3B encodes a low-affinity receptor for the Fc portion of IgG and is expressed almost exclusively on neutrophils (25). It plays a critical role in NET activation (26). Genome-wide association studies have identified FCGR3B copy number variations as being associated with UC susceptibility in Japanese populations (27). Additionally, proteome-wide Mendelian randomization analysis has shown that circulating FCGR3B levels are associated with UC risk (28). Our findings further support that FCGR3B expression correlates positively with UC disease activity and is linked to non-responsiveness to golimumab and infliximab. Therefore, FCGR3B holds potential as a diagnostic biomarker and a therapeutic target in UC.

IL1RN, a member of the interleukin 1 cytokine family, functions to suppress the activity of interleukin 1 alpha (IL1A) and interleukin 1 beta (IL1B), thereby modulating a broad range of interleukin 1-mediated immune responses and promoting NET formation (7, 29). Knockout studies have shown that *Il1rn*-deficient mice spontaneously develop colitis with high mortality. However, in our study, increased expression of IL1RN was positively correlated with UC severity. A possible explanation for this discrepancy is that genetic variations may impair IL1RN functionality, such that even elevated expression levels fail to suppress inflammation effectively (30, 31).

CXCL8, which encodes interleukin-8 (IL-8), is a key chemokine involved in the recruitment of neutrophils to inflammatory sites through chemotactic signaling (32). In our analysis, CXCL8 expression was positively correlated with UC disease activity. This finding aligns with a study by Skrzypczak-Zielińska et al., which reported that the CXCL8 c.91T allelic variant adversely affects disease progression in patients with IBD (32). CXCL8/IL-8 has been shown to promote NET formation, impair wound healing in type 2 diabetes (33), and contribute to enhanced neutrophil infiltration and NET production in the synovium of rheumatoid arthritis (34). Moreover, therapeutic targeting of CXCL8 reduces neutrophil infiltration, suppresses NET formation, and inhibits tumor progression (35). These findings suggest that CXCL8 exacerbates UC by promoting NET formation. However, the specific mechanisms through which CXCL8 drives NET formation in colitis remain unresolved and warrant further investigation.

S100A8 and S100A9 are calcium- and zinc-binding proteins that primarily function as the heterodimer calprotectin (S100A8/A9). This complex mediates neutrophil chemotaxis and adhesion and plays a crucial role in regulating inflammatory responses (36). Our study demonstrated a strong positive correlation between S100A8/A9 expression in colonic tissue and UC disease activity. Similarly, fecal S100A8/A9 levels are well-established, noninvasive biomarkers for monitoring intestinal inflammation in UC (36). Peptides targeting S100A8/A9 at the intestinal mucosa have been shown to ameliorate colitis in murine models (37). S100A8/A9 also contributes to NET formation by enhancing intracellular reactive oxygen species production in neutrophils, which in turn accelerates peptidylarginine deiminase 4 (PAD4)-mediated NET formation, thereby exacerbating neuroinflammation (38). Therefore, S100A8/A9 may likewise contribute to UC pathogenesis via NET promotion. Nonetheless, further studies are needed to elucidate the precise mechanisms by which CXCL8 and S100A8/A9 drive NET formation in colitis.

To better apply our research findings to clinical practice, we constructed two nomograms (39). The first nomogram, designed for diagnostic purposes, incorporates the expression levels of IL1RN, FCGR3B, S100A9, S100A8, and CXCL8 to effectively distinguish patients with UC from healthy individuals. The second nomogram integrates IL1RN and FCGR3B expression levels to improve predictive accuracy in assessing the likelihood of non-response to anti-TNF-α therapies, including golimumab and infliximab. Although multiple models have been developed to predict the therapeutic response to infliximab, relatively few have focused on golimumab. Our model demonstrates high predictive performance for golimumab response, underscoring its clinical relevance. Although both drugs target TNF-α, differences in their manufacturing processes may account for the observed discrepancies in model performance.

Notably, the predictive accuracy of our model varied across international UC cohorts (GSE12251, U.S. cohort; GSE16879, Belgian cohort) treated with infliximab, suggesting that ethnic or regional differences may influence treatment outcomes.

Despite the promising diagnostic and prognostic potential of NET-associated genes in UC, several limitations warrant consideration. The models were developed exclusively using retrospective datasets; thus, prospective validation studies are essential to confirm the reliability. Although we observed strong correlations between NET-associated hub genes and anti-TNF-α treatment failure, further mechanistic studies are required to elucidate how these genes contribute to therapeutic resistance.

In conclusion, NET-associated UC clusters exhibited differential responses to anti-TNF-α therapy. Increased expression of five hub genes was closely associated with disease severity and poor prognosis. Using these genes, we developed a predictive model for identifying patients with UC at risk of non-response to anti-TNF-α treatment. These findings offer valuable insights for improving clinical management and guiding therapeutic decision-making in UC.

## Data Availability

The original contributions presented in the study are included in the article/**Supplementary Material**. Further inquiries can be directed to the corresponding authors.
