# Supplementary material for: Developing models for the diagnosing of ulcerative colitis and prognosis of anti-TNF-α non-response based on neutrophil extracellular trap-associated genes
Source: Front Immunol. 2025 Sep 2;16:1530508. doi: 10.3389/fimmu.2025.1530508 (PMC12436428; doi:10.3389/fimmu.2025.1530508)
Supplement: Supplementary file 1 [file Table1.docx]

Supplementary Material

## Supplementary Figures Legend


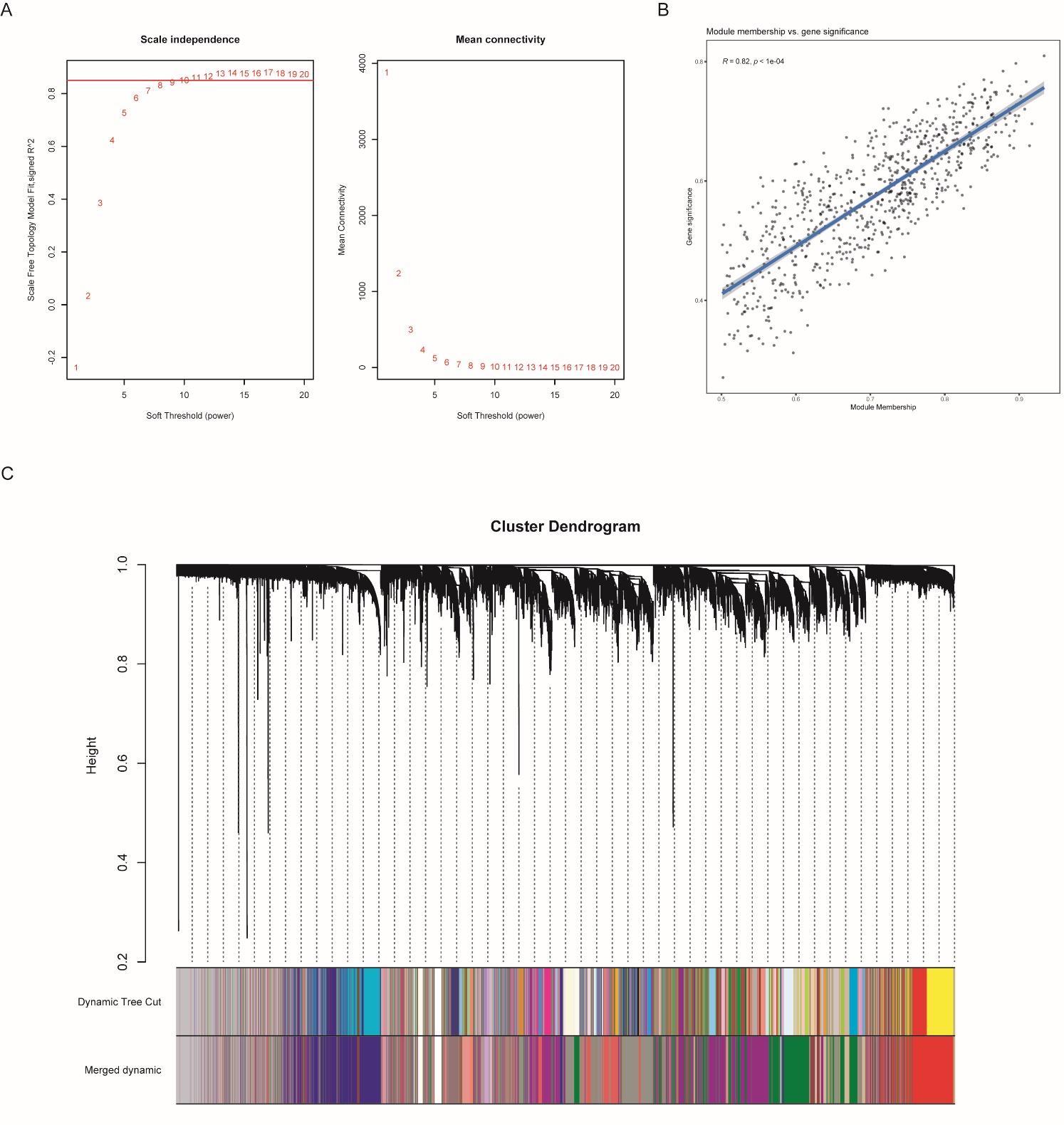


**Supplementary Figure 1. Establishment of a gene co-expression network.(A)**The left panel is configured with a scale-free topological fit index R2 set to 0.85, and an optimal soft threshold β of 10 is selected to achieve the optimal average connectivity for the co-expression network displayed on the right panel. **(B)** A scatter plot illustrates the relationship between brown module membership and UC gene significance, exhibiting a correlation coefficient of cor=0.82 (p<1.4e−4) between them.**(C)**clustering tree of samples, depicting the modules both before and after their merging process, utilizes Dynamic Tree Cut for initial module segmentation and Merged dynamic approach for the outcome of merging highly correlated modules.
